# How should we report the foveal status in eyes with “macula-off” retinal detachment?

**DOI:** 10.1038/s41433-022-02074-7

**Published:** 2022-05-03

**Authors:** Julian E. Klaas, Jakob Siedlecki, David H. Steel, D. Alistair H. Laidlaw, Siegfried Priglinger

**Affiliations:** 1 Department of Ophthalmology, University Hospital, LMU Munich, Munich, Germany; 2 Ophthalmology, Sunderland Eye Infirmary, Sunderland, UK; 3 Guy’s and St. Thomas’ NHS Foundation Trust, London, UK

**Keywords:** Predictive markers, Retinal diseases

## Abstract

Whilst pre- and postoperative multimodal imaging technologies including optical coherence tomography (OCT) have investigated the morphological correlates of worsened visual outcomes in rhegmatogenous retinal detachment (RRD) with foveal involvement, the nomenclature has adhered to the traditional ophthalmoscopy-based and rather vague term “macula-off”. This article appraises the current literature with regard to the preoperative assessment and nomenclature of the foveal status in macula involving retinal detachment (MIRD). A literature review of recent publications assessing functional or morphological outcomes in MIRD was conducted, using the search terms “fovea-off” or “macula-off”. The search date was April 28^th^, 2021. Original studies in English language were included. Case reports, review articles or letters were excluded. Forty relevant articles (range of publication dates: July 29^th^, 2020 - April 18^th^, 2021) were reviewed to assess the diagnostic modalities used, morphological parameters assessed, and any specific nomenclature introduced to specify the extent of macular detachment. The results suggest widespread variability and inconsistencies with regard to the preoperative assessment, diagnostic modalities and nomenclature used to describe the foveal status in eyes with RRD termed “macula-off”. The extent of macular detachment may be classified by a wide range of morphological parameters, including the height of foveal detachment and the ETDRS grid as overlay tool in OCT devices. There is a scientific and clinical need for an updated nomenclature for eyes with “macula-off” RRD. Preoperative OCT findings should be reported on a regular and standardized basis in order to establish a consensus how to report the foveal status in eyes with MIRD.

## Abstract (zh)

尽管多模式影像学技术包括相干光断层扫描 (OCT) 在术前和术后提供了黄斑区受累的孔源性视网膜脱离 (RRD) 所导致的视力恶化的形态学相关性, 但其命名仍遵循传统的基于检眼镜的术语, 且含义相当模糊, 即“黄斑脱离”。本文综述了黄斑中心凹状态在累及黄斑区的视网膜脱离 (MIRD) 中的术前评估与术语命名。本文使用检索词“fovea-off”或“macula-off”对近年来评估MIRD功能或形态学结果的文献进行了综述。文献检索的日期为2021年4月28日, 纳入英文原著, 并排除病例报告、综述文章或信函。本文对40篇相关文章 (发表日期为2020年7月29日 — 2021年4月18日) 进行了综述, 评估文献中使用的诊断方式、形态学参数以及所有引入的用于说明黄斑脱离程度的特定命名法。研究结果表明, 在术前评估、诊断方式和用于描述RRD (称为“黄斑脱离”) 患眼中心凹状态的命名法存在广泛变异性和不一致性。黄斑脱离的程度可通过广泛的形态学参数进行分类, 这些参数包括黄斑中心凹脱离的高度和OCT中作为覆盖工具的ETDRS网格。因此, 科学研究和临床上需要更新“黄斑脱离”RRD患眼的命名。学者们应定期和标准化报告术前OCT结果, 以便就如何报告MIRD患眼的黄斑中心凹状态达成共识。

## Introduction

Despite the advancements in retinal surgery over the past decades, foveal involvement in rhegmatogenous retinal detachment (RRD) is still associated with disappointing visual outcomes, even after prompt and successful retinal surgery [1–3]. Numerous studies and database analyses have assessed a variety of prognostic factors for visual recovery in “macula-off” rhegmatogenous retinal detachment, including individual factors (e.g. age, duration of symptoms, pre-existing retinal pathologies), peri-operative factors (e.g. time and type of surgery, experience of surgeon, follow-up and positioning of patients) as well as anatomical features on first presentation (e.g. foveal involvement, extent of detachment) [2, 4–10]. The purpose of this review was to explore inconsistencies with regard to the nomenclature and assessment of the preoperative macular status in eyes termed “macula-off” in current clinical and scientific practice, and to detail possible morphology-based methods to report the foveal status in macula involving retinal detachment.

## Methods

A literature search was made on Pubmed with the search terms “fovea-off” or “macula-off” on April 28^th^, 2021. Original studies from 2000–2021 were included. The search was restricted to articles written in the English language. Case reports, reviews or letters were excluded. First, relevant articles were reviewed for the assessment of the correlation between preoperative morphology and postoperative functional outcome. As a second step, forty recent articles, published from July 29th, 2020 to April 18th, 2021, were reviewed to assess the diagnostic modalities used, morphological parameters assessed, and any specific nomenclature introduced to specify the extent of macular detachment.

## Preoperative optical coherence tomography in eyes with “macula-off” retinal detachment

Today, as compared to the age of purely observational ophthalmoscopy, the *preoperative* examination of the macula is currently undergoing fundamental change [11, 12]. Multimodal imaging techniques, including high resolution optical coherence tomography (OCT) have been increasingly used to describe the foveal status in eyes previously termed “macula-off” in a more detailed manner, successfully correlating preoperative morphology with postoperative functional outcomes and retinal morphology since their introduction to clinical ophthalmology in 1995 [13]. In 2000, Hagimura et al. reported significant intraretinal changes in one of the first studies examining the detached macula with preoperative OCT [14]. Three preoperative factors were found to correlate significantly with postoperative visual acuity in their cohort of 25 consecutive patients: intraretinal splitting (cystoid cavities), intraretinal splitting with an “undulated” outer retina, as well as the height of foveal detachment [15]. In 2005, Lecleire-Collet et al. suggested that the height of retinal detachment may be a better prognostic indicator than preoperative visual acuity, especially in eyes with lower amounts of subretinal fluid [16]. This study of 20 patients found a highly significant correlation with postoperative visual acuity for the distance between the foveal centre to the nearest undetached retina, especially when combined with intraretinal structural changes [16]. At the same time, Ross et al. were the first to measure the height of macular detachment with 3-dimensional B-scan-ultrasonography in a prospective study of 52 eyes with RRD of less than 7 days duration, and found that shallower subretinal fluid was correlated with better functional outcomes [17]. More recently, in a retrospective review of 180 eyes with RRD and an OCT-based preoperative diagnosis of a detached fovea, Park et al. found that an intact external limiting membrane and the involvement of fewer quadrants were both associated with a better functional recovery [2]. In 2021, Hostovsky et al. published a consecutive case series of 47 eyes with an OCT-based diagnosis of “macula-off” and found that the presence of a macular hole, an epiretinal membrane and the height of foveal detachment correlated to postoperative visual acuity [18]. Further examples of studies assessing preoperative parameters in eyes with “macula-off” are included in Table 1 [12, 19–55].

**Table 1:** Most recent publications involving search keywords “macula-off” or “fovea-off” and their approaches to the nomenclature and assessment of the preoperative macular status in eyes with rhegmatogenous retinal detachment. Search date: April 28th, 2021.

Reference No.	Author (et al.)	Assessment of the macular status with preoperative oct^a^	Specification of the extent of macular detachment^a^	Method to assess the extent of macular detachment	Diagnostic modality used to assess the extent of detachment	Specific nomenclature to distinguish extent of detachment
[19]	Angermann R	yes	yes	submacular fluid volume	oct	–
[20]	Lu B	yes	no	–	–	–
[21]	Chua J	no	no	–	–	–
[22]	Soares RR	no	yes	>50% detachment	not specified	–
[23]	Lee CS	no	yes	quadrants	not specified	–
[24]	Boden KT	yes	yes	ETDRS grid, height of detachment	oct	Grade 1–4 (Zone 1–3)
[25]	Guan I	yes	yes	height of detachment	oct	–
[26]	Ersoz M	yes	yes	not specified	–	–
[4]	Klaas JE	yes	yes	ETDRS grid, height of detachment	oct	MIRD, CIRD (Grade 1–5)
[27]	Christou E	no	yes	quadrants	fundoscopy	–
[28]	Long K	yes	no	–	–	–
[29]	Baudin F	yes	yes	quadrants, height of detachment	fundoscopy, oct	–
[30]	Chatziralli I	yes	no	–	–	–
[31]	Safadi K	no	no	–	–	–
[18]	Hostovsky A	yes	yes	height of detachment	oct	–
[32]	Pole C	no	no	–	–	–
[33]	Chatziralli I	no	no	–	–	–
[34]	Singh A, Behera UC	no	no	–	–	–
[35]	Iwase T	yes	yes	height of detachment, anterior protrusion, angle of detachment	oct	–
[36]	Moussa G	no	no	–	–	–
[37]	Deiss M	yes	yes	clock hours, height of detachment	fundoscopy, oct	FiRD
[38]	Guber J	no	no	–	–	–
[12]	Barca F	yes (+ oct-a)	no	–	–	–
[39]	Konstantinidis L	yes	yes	height of detachment	oct	–
[40]	Guber J	no	yes	quadrants	not specified	–
[41]	Poyser A	no	no	–	–	–
[42]	Kaderli ST	yes (+ oct-a)	no	–	–	–
[43]	Maqsood S	no	no	–	–	–
[44]	Fu Y	yes	yes	quadrants	not specified	–
[45]	Jasani KM	partially	no	–	–	–
[46]	Degenhardt V	yes	no	–	–	–
[47]	Ng H	yes	yes	quadrants	not specified	–
[48]	Yeo Y	yes	yes	quadrants	not specified	–
[49]	Abouhussein M	partially	no	–	–	–
[50]	Liu R, Li Q	no	no	.	–	–
[51]	Patel S	partially	yes	clock hours, foveal vs macular detachment	fundoscopy, oct	Macula-Split, Macula-Threatening
[52]	Ng H	yes	yes	ETDRS grid	oct	Stage 1 - Stage 6
[53]	Kosacki J	yes	yes	height of detachment	oct	–
[54]	Mané V	yes	yes	distance to fovea	oct	–
[55]	Chatziralli I	yes	no	–	–	–

*oct* optical coherencetomography, *oct - a* optical coherence tomography angiography, *ETDRS* Early Treatment Diabetic Retinopathy Study, *FiRD* Fovea Involving Retinal Detachment, *MIRD* Macula Involving Retinal Detachment, *CIRD* Center Involving Retinal Detachment.

^a^no = no or not otherwise specified.

While the correlation between worsened functional outcomes and retinal morphology has been described in unprecedented detail by modern multimodal imaging devices, the relationship between photoreceptor damage and retinal morphology has already been investigated thoroughly by Machemer et al. more than 50 years ago in multiple histologic, phase-contrast and electron microscopic autoradiographic studies of experimental retinal detachments in owl monkeys [56]. Thus, many of the aforementioned morphological features of the detached and reattached retina seem to affirm and correspond to the profound knowledge gained through these early experimental studies, including the time and pattern of photoreceptor renewal after retinal reattachment and the recovery of ERG responses [57–59]. Remarkably, even though the correlation between preoperative morphology and postoperative visual function has been demonstrated repeatedly, no consensus on the preoperative assessment of the macular status in eyes termed “macula-off” has yet been established. As a result, retina specialists are confronted with a multitude of studies, which – even though mostly well-executed – often assess a wide range of non-standardized parameters in non-standardized ways. This variability limits the comparability between studies and poses a major question to the scientific and clinical community: Do we still speak the same language when we say “macula-off”, - or in other words - can we do better in classifying and reporting the foveal status of eyes with an ophthalmoscopy-based diagnosis of “macula-off”?

## Literature review of the nomenclature and assessment of eyes with “macula-off”

Anatomically, the term *macula* refers to the central area of the retina located in between the temporal vascular arcades and the optic nerve head, measuring roughly 5.5 mm in diameter [60]. The *fovea*, the *foveola* and its central plateau (called the *umbo*) measure approximately 1.5 mm, 0.3 mm and 0.15 mm in diameter, respectively. While most retina specialists may have accepted the term “macula-off” as a sufficient means to describe a detached *foveal* status, a review of 40 recent publications focusing on “macula-off” retinal detachment (search term “macula-off” or “fovea-off” on pubmed.gov, date April 28^th^, 2021, Table 1) highlights that the term “macula-off” may include a variety of morphological states which, - depending on the degree of detail, – may be based on different diagnostic methodologies, ranging from fundoscopy-based approaches (e.g. “clock hours”, “quadrants”) to grid-based strategies using preoperative OCT, albeit comparatively rarely [1, 2, 24, 52, 61, 62]. The inconsistency in assessing and reporting the macular status, as is evident in the up-to-date scientific literature is even more surprising with the continuous technical improvements of OCT devices over the past two decades, especially in high detachments [18]. The ability of newer generation OCT scanners to image the structure of a detached macula may be under appreciated: a development which may provide a much greater understanding of the aetiology, prognosis and recovery of vision following “macula-off” retinal detachment. Names matter, both in a clinical context as well as scientific publications, which is why frequently-used terms need to be regularly revised and if necessary adapted to newer examination modalities—a process reminiscent of the once inconsistent and non-standardized approach to classification of diffuse versus focal diabetic macular oedema [63]. Only once we have reached consensus as to what morphological situation we are referring to when we talk about “macula-off”, can we advance our art, and add to the evidence-base of RRD with macular and foveal involvement. Ultimately, a universal morphology-based classification of the preoperative macular status may not only facilitate communication between retina specialists and their fellows (e.g., over the timing of surgery in dependence of distance to fovea) but also with our patients, guiding us in determining individual outcomes and optimal treatments.

## The need for a uniform morphology-based nomenclature

**Fig. 1:**
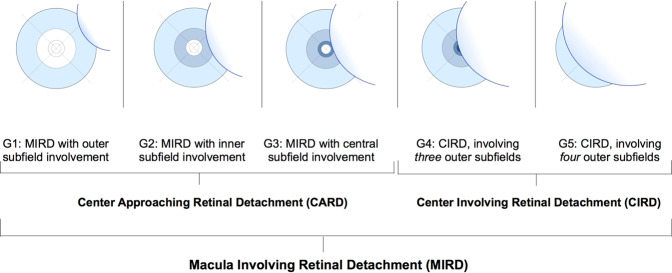
Proposal of a five-tier grading system for macula involving retinal detachment. Visualisation and nomenclature of five grades of Macula Involving Retinal Detachment (MIRD) based on the morphological extent of involvement using an ETDRS-grid overlayed on a 30° Infrared-Image and the corresponding optical coherence tomography scan, adapted from Klaas et al. [4]. Grade 4 (G4) and Grade 5 (G5) are distinguished by a detached foveal centre and are referred to as Center Involving Retinal Detachment (CIRD). In contrast, a center approaching situation (G1-G3) is labelled as Center Approaching Retinal Detachment (CARD).

**Fig. 2:**
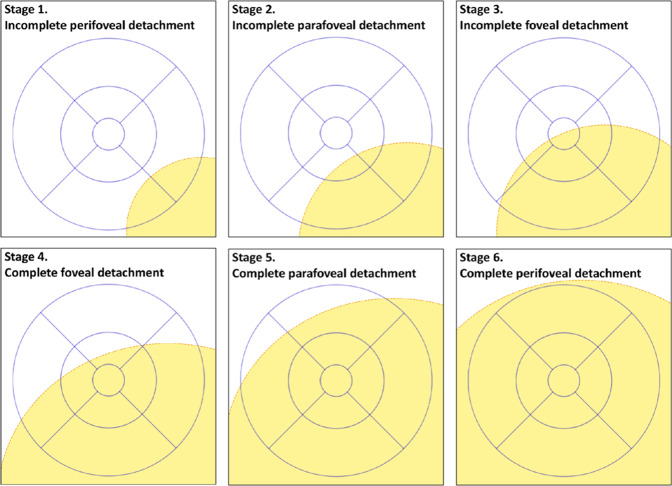
Proposal of a six-tier grading system for macula involving retinal detachment. Visualisation and nomenclature of a six-tier grading system for the detached macula using the ETDRS grid as proposed by Ng et al. [52].

**Fig. 3:**
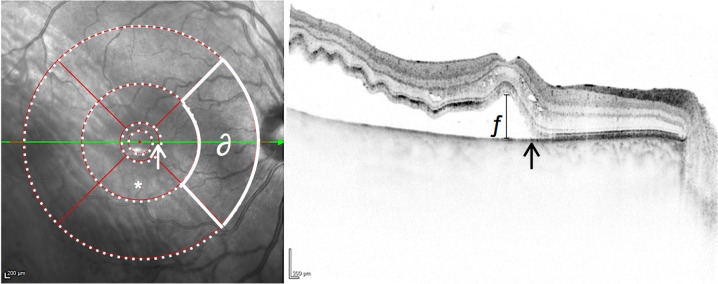
Example of retinal detachment involving the foveal center and three outer subfields. Infrared-Image with ETRDS grid overlay in Heyex-2-Software (Heidelberg Engineering, Heidelberg, Germany) and SD-OCT (30°), representing Centre Involving Retinal Detachment with three outer subfield involvement (CIRD G4). Dashed white circles  are added to indicate subretinal fluid within this part of the ring. A continuous white line indicates that subretinal fluid does not cross this boundary. The white and black arrows both mark the margin of detachment in the IR and OCT image, respectively. In at least one outer subfield (∂) no subretinal fluid can be detected in volume or radial scans. * marks one of the four inner (parafoveal) subfields of the ETDRS grid. ƒ marks height of foveal detachment (=291 µm, as measured perpendicularly to the RPE in 1:1 resolution).

**Fig. 4:**
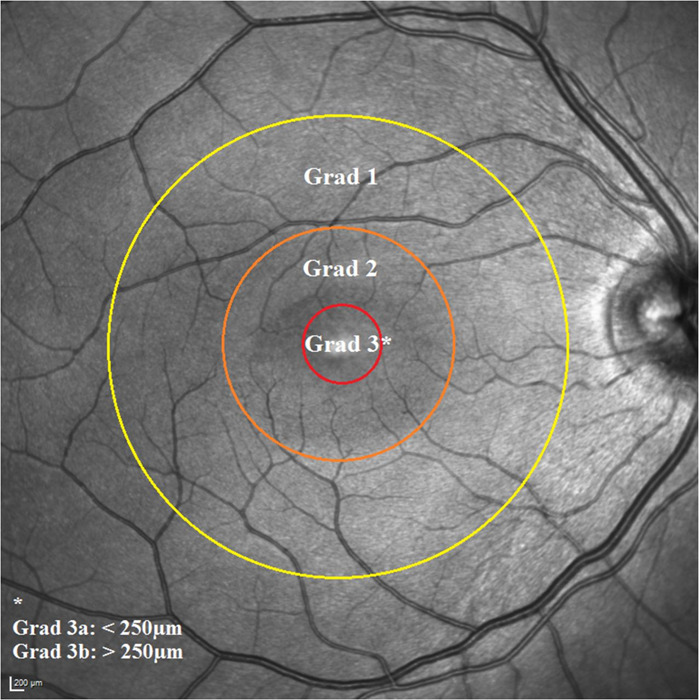
Proposal of a three-tier grading system for macula involving retinal detachment. Visualisation of a grid-based grading system for eyes with macula involving retinal detachment using the 1.0 mm (red), 3.0 mm (pink) and 6.0 mm (yellow) diameter of the ETDRS grid as proposed by Boden et al. [24]. Grade 3 is further subdivided according to the height of foveal detachment in the corresponding OCT scan (cutoff = 250 µm).

But how can we report the foveal status more accurately? Above all, a morphology-based nomenclature should be easy to understand and apply on the one hand and improve outcome prediction on the other hand. Thus, a potential classification could possibly benefit from prior endeavours to correlate retinal morphology and function. We believe that a commonly accessible and easy-to-apply tool such as the ETDRS-grid could meet the need for an outcome-related grading of the macular status, as presented at the 18^th^ Congress of the European Society of Retina Specialists [64]. Our proposed scheme (Fig. 1) classifies five grades of macula involving retinal detachment (MIRD) based on the extent of macular involvement in preoperative OCT, adhering to the original ETDRS nomenclature (centre = centre of the central subfield) [65]. In 2020, Ng et al. adapted the aforementioned scheme in a prospective study of 48 patients, illustrating and expanding its applicability (Fig. 2) [52]. In a retrospective cohort of 102 eyes with MIRD, we found that a detachment involving four outer subfields of the grid (CIRD G5, Fig. 1) was correlated with a significantly worse postoperative visual acuity in comparison to a foveal detachment involving only three outer subfields (CIRD G4, Fig. 1 and Fig. 3) [4]. Boden et al. have suggested combining the height of foveal detachment with the extent of detachment in a further ETDRS-grid-based grading scheme in 108 eyes with macula involving retinal detachment (Fig. 4) [24]. But what if we cannot assess the height of detachment due to high detachment of the macula by OCT? Even though the limited axial imaging range of OCT devices (~2000 µm) can make imaging of bullous RRDs impossible, techniques such as consecutive axial scanning have been proposed [16]. Ultimately, however, precise imaging may be more important in eyes with a *lower* amount of subretinal fluid and a *lower* extent of detachment that could benefit most from earlier surgery. This could prevent further structural damage, visualised through morphological changes in the preoperative OCT, such as the formation of cystoid cavities or early outer retinal atrophy [2, 4, 24, 66].

## Conclusion

The data and different solutions presented herein demonstrate that the variety of morphological phenotypes and outcomes witnessed in eyes with macula involving RRD may not be met sufficiently by the established names “macula-off” vs. “macula-on” anymore. Instead, there is a growing scientific and clinical need for an updated more precise nomenclature, as it could be assessed routinely using optical coherence tomography *before* surgical intervention.

In conclusion, we believe future studies should be initiated on the basis of an international consensus, recommending how to report the foveal status in eyes with macula involving RRD. Commonly available overlay tools, such as the ETDRS-grid in OCT devices have been shown to be of use in grading the preoperative extent of detachment and may help in assessing additional morphological features in a standardized manner, which in turn may be integrated into a future classification system. In doing so, we may shed a brighter light on how a possible *and* probable path to recovery after macula involving RRD may look like, hence reaching an evidence- and morphology-based agreement on both the immediacy of our management *and* the patient’s individual risk for long-term vision-loss.

## Data Availability

This article includes data accessed via https://pubmed.ncbi.nlm.nih.gov on April 28th, 2021. Each article was downloaded via an institutional access. The data that support this article including the studies cited are available on https://pubmed.ncbi.nlm.nih.gov using terms “fovea-off” OR “macula-off”.
